# Acute liver abscess after non-operative management of blunt liver injury: A rare case managed with laparoscopic drainage

**DOI:** 10.1016/j.ijscr.2020.04.092

**Published:** 2020-05-14

**Authors:** Mu-Yun Lin, Ching-Yun Liao, Being-Chuan Lin

**Affiliations:** a Chang Gung University College of Medicine, Tao-Yuan City, Taiwan; b Chang Gung University School of Traditional Chinese Medicine, Tao-Yuan City, Taiwan; c Division of Trauma & Emergency Surgery, Department of Surgery, Chang Gung Memorial Hospital, Chang Gung University, 5, Fu-Hsing Street, Kwei-Shan, Tao-Yuan City, 333, Taiwan

**Keywords:** Non-operative management, Blunt liver injury, Liver abscess, Laparoscopic drainage

## Abstract

**Introduction:**

Liver abscess may develop as a rare complication of the non-operative management (NOM) of blunt liver injury.

**Presentation:**

A 36-year-old male was injured in a motorcycle accident on November 28, 2017. First aid was performed at the local hospital, then he was transferred to our trauma center for further management. The abdominal computed tomography (CT) revealed a segment 7/8 liver laceration, and the liver injury was of grade III according to the American Association for the Surgery of Trauma-Organ Injury Scale for liver injury. Intermittent high fever was observed for the first 3 days after NOM, and repeat abdominal CT showed an abscess with rupture at the previously injured liver parenchyma. He underwent laparoscopic drainage of the liver abscess, and culture revealed the presence of Salmonella enterica, serogroup D. After laparoscopic drainage, the patient recovered well, with a 21-day hospital stay.

**Discussion:**

Liver abscess as a complication after NOM of blunt liver injury is a rare entity, with an incidence rate of 1.5%. It is usually seen in major liver injuries (grade III and above) and the abscesses take a median of 6 days (range, 1–12 days) to form and be diagnosed. The management of liver abscess may be by surgical drainage (laparotomy or laparoscopy) or percutaneous drainage.

**Conclusion:**

This report reminds us the liver abscess complication after NOM of blunt liver injury, although it is a rare entity. Results of this patient support drainage of the liver abscess can be safely and effectively performed by laparoscopy.

## Highlights

• Non-operative management (NOM) has today become the first treatment of choice when possible in patients with blunt liver injury.
• Liver abscess as a complication after NOM of blunt liver injury is a rare entity, with an incidence rate of 1.5%.
• The most common bacteria responsible for liver abscess include Klebsiella pneumoniae, Staphylococcus aureus, Streptococcus pyogenes, gram-positive cocci, Clostridium, and mixed organisms.
• Laparoscopic drainage can be performed safely and effectively for the liver abscess.

## 1 Introduction

Non-operative management (NOM) has today become the first treatment of choice when possible in patients with blunt liver injury. Complications of NOM of blunt liver injury are rare, but may include biloma, hepatic artery pseudoaneurysm, liver necrosis, liver abscess, and delayed hemorrhage [[1], [2], [3]]. Liver abscess is a rare complication after NOM and management of this complication with laparoscopic approach is seldom reported. We report a case that developed liver abscess acutely after NOM of blunt liver injury and was managed successfully with laparoscopic drainage. The work has been reported in line with the SCARE guidelines [4].

## 2 Case presentation

**Fig. 1:**
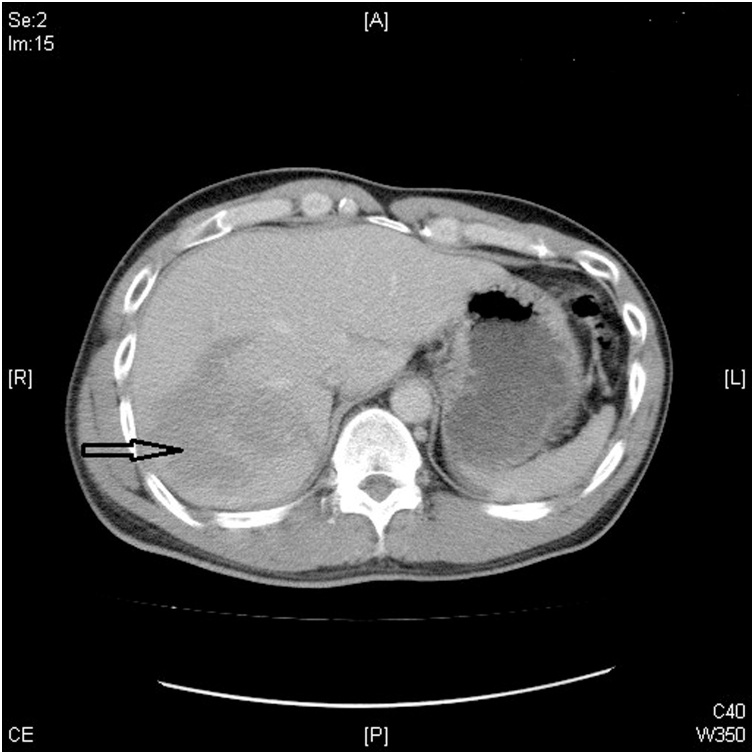
Abdominal computed tomography scan of the 36-year-old man, showing intra-parenchymal laceration with hematoma (arrow).

**Fig. 2:**
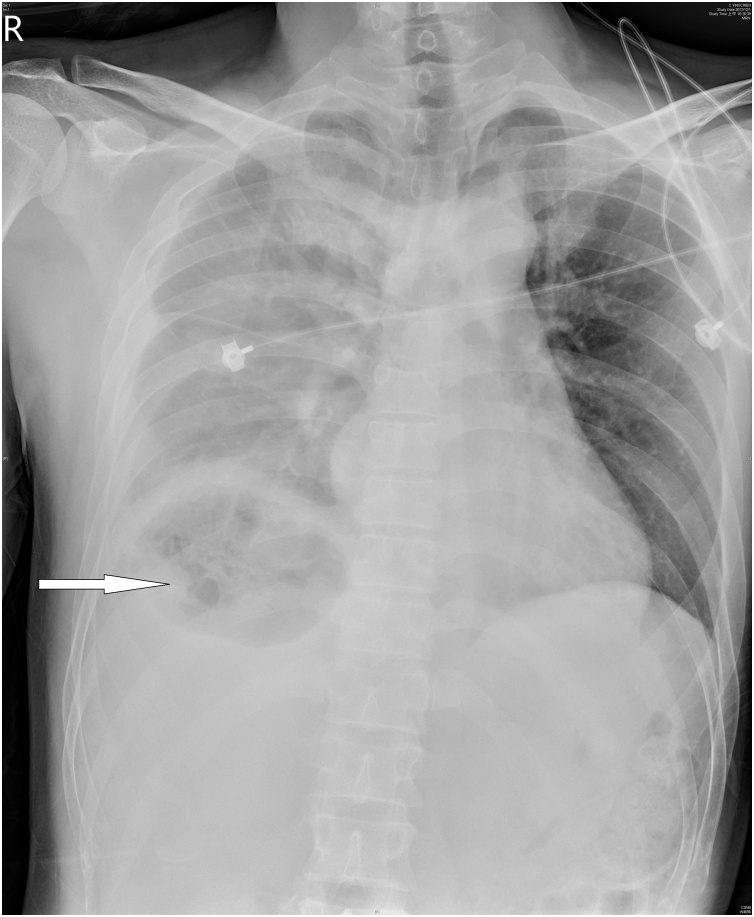
Chest radiography of the same patient performed 58 h after first abdominal computed tomography scan, showing right subphrenic abnormal entrapped air (arrow).

**Fig. 3:**
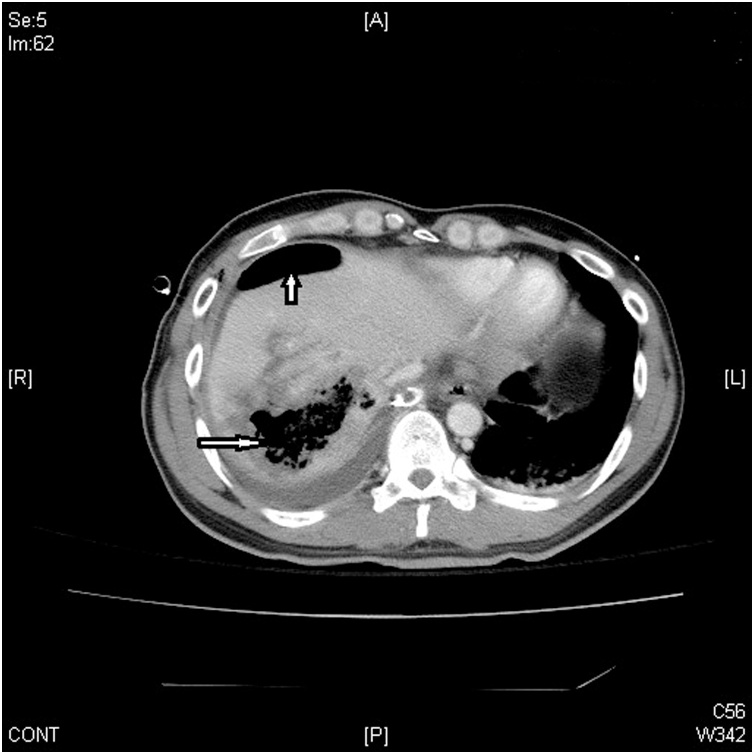
Abdominal computed tomography scan of the same patient performed 4 h after chest radiography, showing a gas-containing liver abscess over the previous injured liver (long arrow). The perihepatic fluid accumulation and intra-peritoneal free air indicated rupture of abscess (short arrow).

**Fig. 4:**
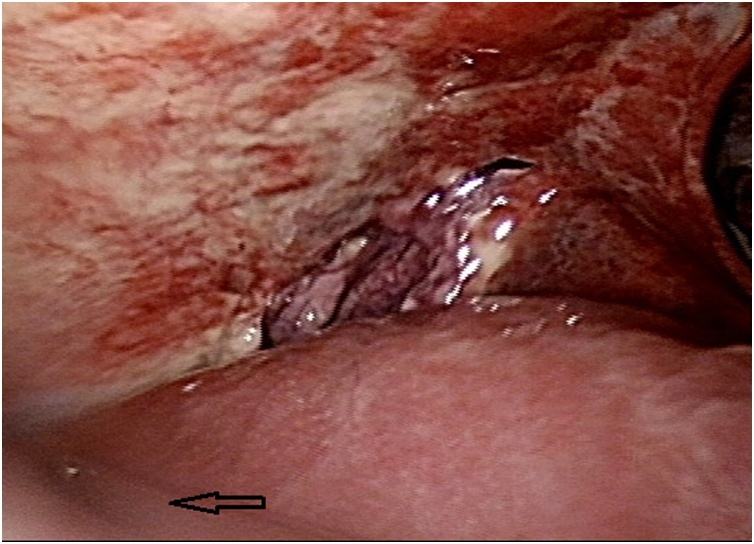
Laparoscopic view of the same patient, showing perihepatic turbid bloody fluid accumulation (arrow).

**Fig. 5:**
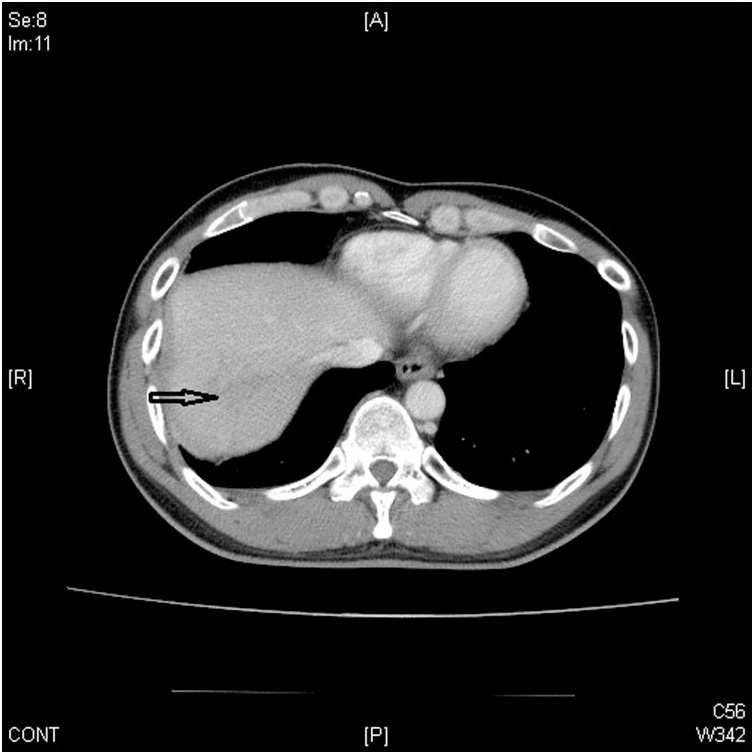
Abdominal computed tomography scan of the same patient performed 4 months after surgery, showing a well-healed liver injury and no residual abscess (arrow).

A 36-year-old man, who was a chronic hepatitis C carrier presented at a local hospital after a motorcycle accident on November 28, 2017. First, resuscitation was performed and abdominal computed tomography (CT) revealed a liver injury. Then, he was transferred to our emergency department (ED) after resuscitation. At the ED, he presented a full Glasgow coma scale and his blood pressure was 118/82 mmHg, respiratory rate was 20 breaths/min, and pulse rate was 65 beats/min. Right upper abdominal tenderness was noted on physical examination, and blood tests revealed a white blood cell count of 9200/mm^3^, hemoglobin level of 13.4 gm%, serum aspartate aminotransferase level of 1165 U/L, and serum alanine aminotransferase level of 1173 U/L. On reviewing the abdomen computed tomography (CT) scan, a grade III liver injury was observed, in accordance with the American Association for the Surgery of Trauma–Organ Injury Scale for liver injury (Table 1) [5]. The segment 7/8 of the liver showed a large intra-parenchymal hematoma, and there was no hemoperitoneum or contrast extravasation (Fig. 1). Thus, he was admitted to the intensive care unit (ICU) for NOM of the blunt liver injury. In the ICU, intermittent high fever up to 38.7 °C was observed, which lasted for three days, and chest radiography revealed right pleural effusion and some entrapped air over the right subphrenic space (Fig. 2). Then, repeat abdominal CT on the same day revealed a large abscess with rupture at the previously injured liver parenchyma with air and fluid accumulation around the perihepatic space (Fig. 3). Then, laparoscopic drainage of the ruptured liver abscess was performed immediately. During the surgery, the surgeon and the camera operator stood on the patient’s left side, with an assistant on the other side. Carbon dioxide pneumoperitoneum (12 mmHg) was established through an umbilical incision using Hasson technique, and a zero-degree-angled laparoscopy was introduced through an 11-mm port. Then, a 5 mm port for liver access was placed at the left lateral abdomen, and a third 5 mm port was placed at the right lateral abdomen to elevate the liver. By laparoscopy, the turbid bloody fluid and necrotic tissue around the injured liver could be drained well (Fig. 4). Approximately 250 mL of infective bloody fluid was drained and sent for culture. Finally, a chest tube was placed for drainage via the right-side port wound. After laparoscopic drainage, fever subsided and the abscess culture revealed the presence of *Salmonella enterica*, serogroup D. According to the sensitivity test, ertapenem (Merck Sharp & Dohme, France) 1 g QD intravenously was given for 11 days. He recovered well and was discharged on December 15, 2017. Follow-up abdominal CT performed 4 months later showed a well-healed liver injury and no residual abscess (Fig. 5).

**Table 1:** Liver Injury Scale (2018 revision).

AAST Grade	Type of injury	Imaging Criteria (CT Findings)
I	Hematoma	subcapsular, <10% surface area
	Laceration	parenchymal, <1 cm parenchymal depth

II	Hematoma	subcapsular, 10–50% surface area
	Hematoma	intraparenchymal, <10 cm diameter
	Laceration	1–3 cm parenchymal depth, <10 cm length

III	Hematoma	subcapsular, >50% surface area; ruptured subcapsular or parenchymal hematoma
	Hematoma	intraparenchymal, >10 cm
	Laceration	>3 cm parenchymal depth
	Any injury in the presence of a liver vascular injury or active bleeding contained within liver parenchyma	

IV	Laceration	parenchymal disruption involving 25–75% hepatic lobe or involves 1–3 Couinaud segments
	Active bleeding extending beyond the liver parenchyma into the peritoneum	

V	Laceration	parenchymal disruption involving >75% of hepatic lobe
	Vascular	juxtahepatic venous injury to include retrohepatic vena cava and central major hepatic veins

AAST, American Association for the Surgery of Trauma; CT, computed tomography.

## 3 Discussion

Since few decades, non-operative management of blunt liver injuries has been a mainstream for patients who are hemodynamically stable, and the success rates for non-operative management are usually greater than 85% [[1], [2], [3],^6^]. Several contributing factors have been recognized: (1) Realization that more than 50% of liver injuries stop bleeding spontaneously, (2) the precedent successful non-operative management in pediatric patients, (3) knowledge that the liver has tremendous capacity to heal after injury, (4) improvements in liver imaging with CT, and (5) use of adjunctive transarterial embolization (TAE) [7]. Many patients who undergo NOM after liver injury, especially high-grade blunt liver injury [8], have liver-related complications, including bile leaks, hemobilia, liver necrosis, liver abscess, and delayed hemorrhage [[1], [2], [3]]. According to previous data based on the same institution, liver abscess as a complication after NOM of blunt liver injury is a rare entity, with an incidence rate of 1.5% [9]. It is usually seen in major liver injuries (grade III and above) and the abscesses take a median of 6 days (range, 1–12 days) to form and be diagnosed [9]. The most common bacteria responsible for liver abscess include Klebsiella pneumoniae, Staphylococcus aureus, Streptococcus pyogenes, gram-positive cocci, Clostridium, and mixed organisms. In the presented case, the patient developed liver abscess within 72 h of NOM, which is rare. The culture of the body fluid revealed *S. enterica* serogroup D, which is usually found in specific poultry [10]. The management of liver abscess may be by surgical drainage (laparotomy or laparoscopy) or percutaneous drainage. Percutaneous CT-guided drainage is regarded as first-line management in many cases, and may play a role in controlling the infection. Although laparotomy remains an option, lavage and drainage can be safely and effectively performed by laparoscopy [11,12]. Laparoscopic washout of infective blood or bile leak is gaining traction as an adjunctive therapy to non-operative management of liver trauma. Because liver abscess in this patient had already ruptured into the peritoneal cavity, surgical drainage was chosen. In this patient, we chose laparoscopy to manage the liver abscess rather than laparotomy with the expectation of benefit from a shorter hospital stay, lesser postoperative pain, and quicker recovery.

## 4 Conclusion

Acute onset of liver abscess after NOM of a blunt liver injury is rather rare, and laparoscopic drainage can be performed safely and effectively for the liver abscess. Patients may thus benefit from the lesser postoperative pain and quicker recovery.

## Declaration of Competing Interest

Drs Lin BC, Lin MY, and Liao CY have no conflicts of interest or financial ties to declare.

## Funding

There are no sources of funding for this case report.

## Ethical approval

This study was approved by our institutional review board (IRB No.: 201801246B0).

## Consent

Informed consent was obtained by the patient for the publication of this case report and images.

## Author contribution

Lin MY and Liao CY analyzed the patient data and wrote the first draft of this manuscript. Lin BC contributed to the final approval of the version to be published. All authors read and approved the final manuscript.

## Registration of research studies

researchregistry5531.

## Guarantor

Lin BC.

## Provenance and peer review

Not commissioned, externally peer-reviewed.
